# Supplementary material for: Investigation of Antioxidant Activity of Protein Hydrolysates from New Zealand Commercial Low-Grade Fish Roes
Source: Mar Drugs. 2024 Aug 11;22(8):364. doi: 10.3390/md22080364 (PMC11355341; doi:10.3390/md22080364)
Supplement: Supplementary file 1 [file marinedrugs-22-00364-s001.zip › marinedrugs-3146590-supplementary.pdf]

Table S1. Proximate composition (% of wet tissue) of New Zealand Hoki and Gemfish roe.

Items	Moisture	Lipid	Protein	Ash	Carbohydrate
**Hoki roe**	68.8 ± 0.38^{A}	10.1 ± 0.65^{A}	17.9 ± 0.72^{B}	1.3 ± 0.12	1.9 ± 0.83^{B}
**Gemfish roe**	63.2 ± 0.70^{B}	7.6 ± 0.03^{B}	23.8 ± 0.74^{A}	1.3 ± 0.06	4.2 ± 0.64^{A}

Different superscript letters ($^{A, B}$) in the same column indicate a significant difference ($P < 0.05$). Carbohydrate content was determined by subtracting the sum of moisture, protein, lipid, and ash from 100%. Results are presented as mean ± standard deviation.

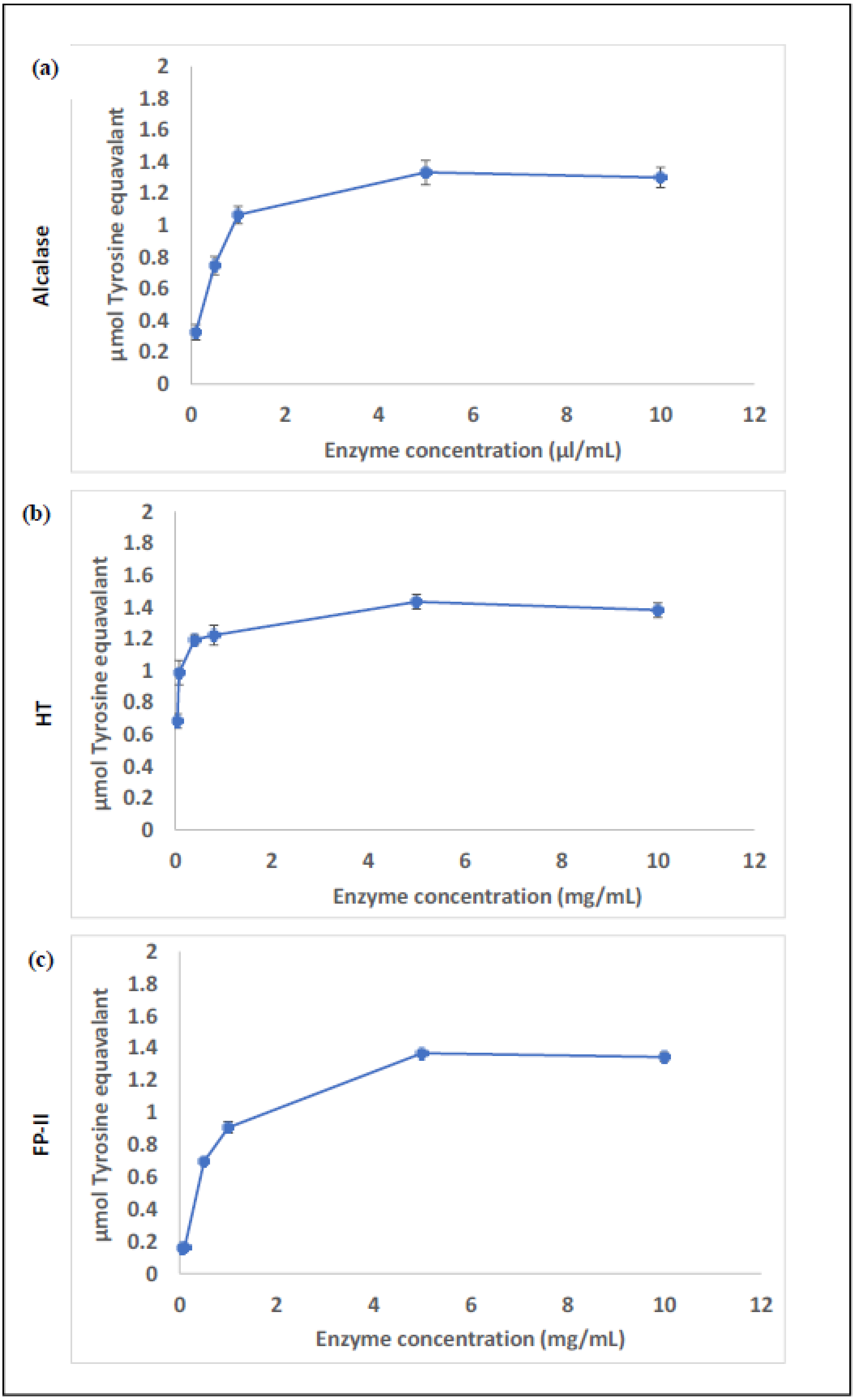

Figure S1. Casein hydrolysis as a function of enzyme hydrolysis using Alcalase (**a**), HT (**b**), and FP-II (**c**).

Protease (Alcalase, HT, and FP-II) hydrolysis at pH (7.0) and temperature (45 °C). The result of umol tyrosine equivalent was applied to indicate the activity of different protease concentration with 30 min hydrolysis. Alcalase was commercially provided as a solution concentration of the prepared stock solution; the enzyme solution was prepared alcalase (µl/mL), HT (mg/mL), and FP-II (mg/mL), as mentioned on the X-axis.

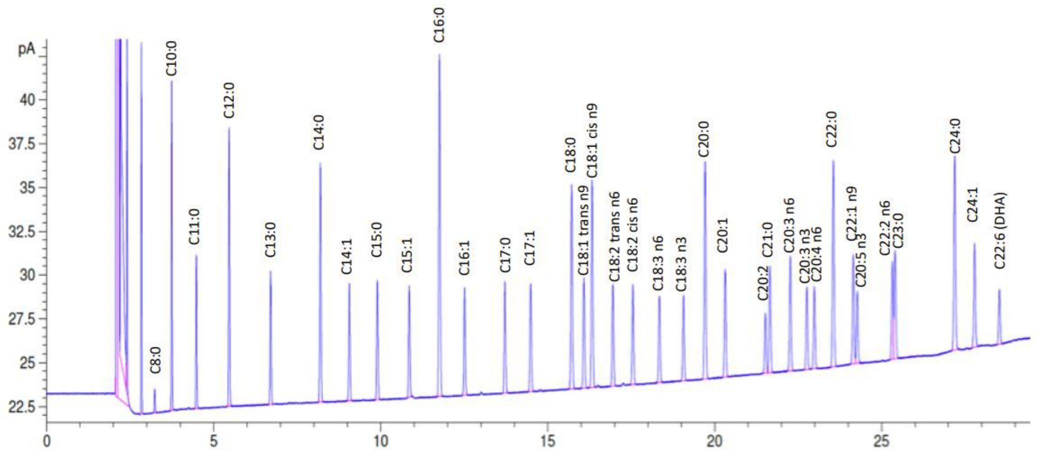

Figure S2. Analysis of commercial FAME standard by GC-FID

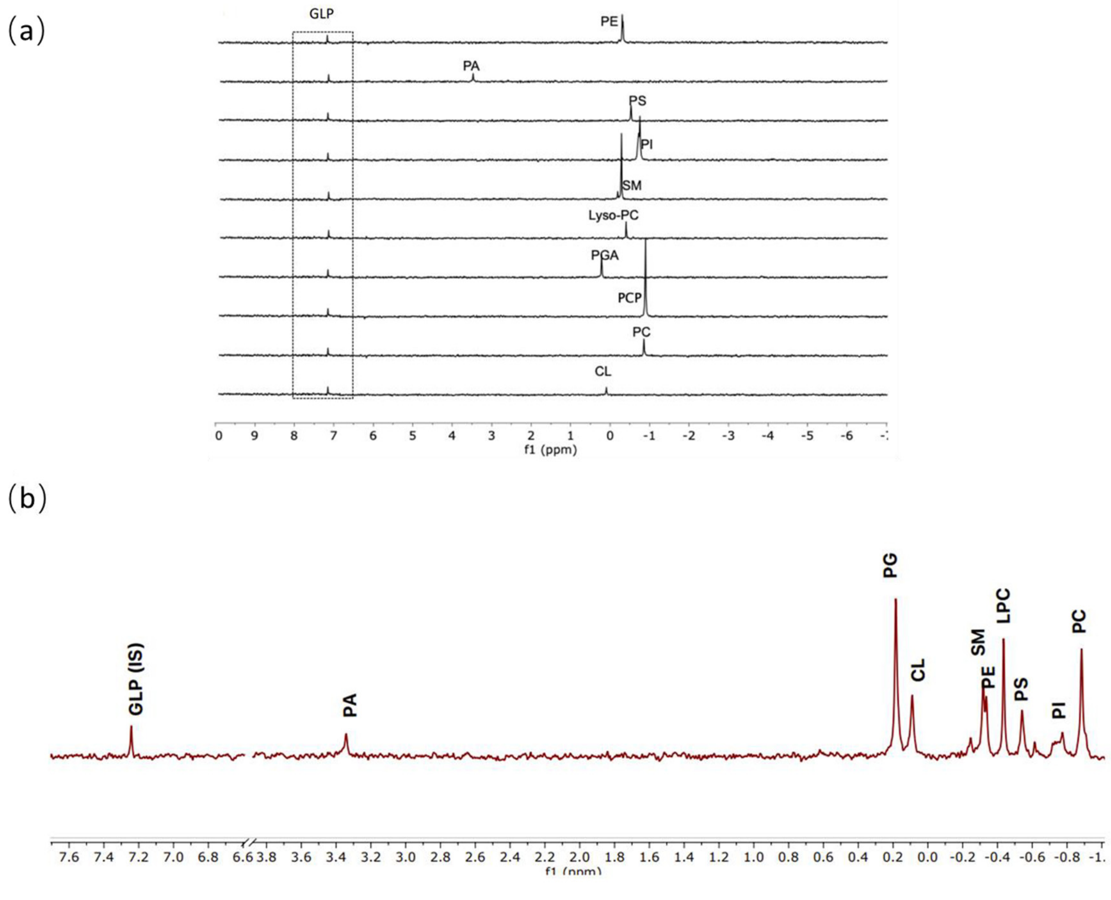

Figure S3. NMR analysis of individual phospholipid standards in D2O (**a**). Standard mix in D2O (**b**).

Abbreviations: PA= phosphatidic acid; PG= phosphatidylglycerol; Cl= cardiolipin; PE= phosphatidylethanolamine; LPC= lyso-phosphatidylcholine; SM= sphingomyelin; PI=

Table S2. Summary of roe homogenate sample and enzyme formulation for time course hydrolysis

	Gemfish roe (*w/w* or *v/w*)	Hoki roe (*w/w* or *v/w*)
Frozen thaw-Alc	2%, 6% and 10%	2%, 6% and 10%
Frozen thaw-HT	2%, 6% and 10%	2%, 6% and 10%
Frozen thaw-FP-II	2%, 6% and 10%	2%, 6% and 10%
Delipidation-Alc	2%, 6% and 10%	2%, 6% and 10%
Delipidation-HT	2%, 6% and 10%	2%, 6% and 10%
Delipidation-FP-II	2%, 6% and 10%	2%, 6% and 10%
Freeze-dried-Alc	2%, 6% and 10%	2%, 6% and 10%
Freeze-dried-HT	2%, 6% and 10%	2%, 6% and 10%
Freeze-dried-FP-II	2%, 6% and 10%	2%, 6% and 10%
Delipidation and Freeze-dried-Alc	2%, 6% and 10%	2%, 6% and 10%
Delipidation and Freeze-dried-HT	2%, 6% and 10%	2%, 6% and 10%
Delipidation and Freeze-dried-FP-II	2%, 6% and 10%	2%, 6% and 10%

-Alc, -HT, -FP-II: sample hydrolysis by protease alcalase, HT, and FP-II.

w/w and *v/w*: weight of protease/ weight of roe homogenate and volume of protease/ weight of roe homogenate.
